# Investigation of Serum Oxidized Low-Density Lipoprotein IgG Levels in Patients with Angiographically Defined Coronary Artery Disease

**DOI:** 10.1155/2014/845960

**Published:** 2014-02-03

**Authors:** Mohsen Moohebati, Vahid Kabirirad, Majid Ghayour-Mobarhan, Habibollah Esmaily, Shima Tavallaie, Amir Akhavan Rezayat, Hossein Pourghadamyari, Amirhossein Sahebkar

**Affiliations:** ^1^Cardiovascular Research Center, Mashhad University of Medical Sciences, Mashhad, Iran; ^2^Department of Cardiology, Mashhad University of Medical Sciences, Mashhad, Iran; ^3^Student Research Committee, Biochemistry of Nutrition Research Center, School of Medicine, Mashhad University of Medical Sciences, Mashhad, Iran; ^4^Biochemistry of Nutrition Research Center, School of Medicine, Mashhad University of Medical Sciences, Mashhad 9177948564, Iran; ^5^Health Sciences Research Center, Department of Biostatistics and Epidemiology, School of Health, Mashhad University of Medical Sciences, Mashhad, Iran; ^6^Department of Clinical Biochemistry, Tehran University of Medical Sciences, Tehran, Iran; ^7^Biotechnology Research Center, Mashhad University of Medical Sciences, Mashhad, Iran

## Abstract

It has been suggested that antioxidized low-density lipoprotein (anti-oxLDL) antibodies play a role in the pathogenesis of atherosclerosis. The aim of this study was to measure serum ox-LDL IgG levels in 31 patients with angiographically defined coronary artery disease (CAD) (≥50% stenosis in at least one major coronary artery; CAD^+^ group) and compare these levels with those of 32 subjects with <50% coronary stenosis (CAD^−^ group) and 24 healthy age- and sex-matched controls using ELISA. We did not find any significant difference between CAD^+^, CAD^−^, and control groups in regard to oxLDL IgG levels (*P* = 0.83). Serum oxLDL IgG levels did not differ between 1VD (one vessel disease), 2VD (2 vessels disease), and 3VD (3 vessels disease) subgroups of CAD^+^ patients (*P* = 0.20). Serum anti-oxLDL titers were only significantly correlated with LDL-C in the CAD^+^ group (*P* < 0.05) and waist and hip circumference (*P* < 0.05 and *P* < 0.01, resp.) in the CAD^−^ group. In stepwise regression analysis, none of the conventional cardiovascular risk factors was associated with serum ox-LDL IgG levels. The present results suggest that serum levels of ox-LDL IgG are neither associated with the presence and severity of CAD nor with the conventional cardiovascular risk factors.

## 1. Introduction

Coronary artery disease (CAD) is a major cause of mortality and disability worldwide [1]. The major independent risk factors for CAD including age, gender, hypertension, cigarette smoking, diabetes mellitus, elevated serum levels of total and low-density lipoprotein cholesterol (LDL-C), and diminished levels of high-density lipoprotein cholesterol (HDL-C) are usually applied for the assessment and management of cardiovascular risk [2]. However, these established risk factors can only explain about 25–30% of the total cardiovascular risk in patients [3], suggesting that other potential factors also play an important role in the pathogenesis of atherosclerosis and CAD. In recent decades, oxidative stress [4], inflammation [5], and immune responses [6] have been considered as emerging risk factors that can significantly contribute to the development of vascular events.

Oxidized low-density lipoproteins (ox-LDLs) are believed to play a pivotal role in atherogenesis [7]. Oxidative modification of LDL is a prerequisite for the accumulation of LDL in macrophages and formation of foam cells. Physicochemical and immunological properties of LDL particles isolated from atherosclerotic lesions have been found to resemble those of ox-LDL [8]. Moreover, immunocytochemical investigations have identified both ox-LDL epitopes and anti-ox-LDL immunoglobulins within atherosclerotic lesions [8–10]. Notably, antioxidant therapy has been shown to reduce atherogenesis in experimental animal models [11, 12]. Antibodies against malondialdehyde- (MDA-) modified LDL have been reported to predict the progression of carotid atherosclerosis [13], CAD [14], and myocardial infarction [15]. Furthermore, results from studies by Heitzer et al. [16] and Raitakari et al. [17] have indicated that antibodies against Cu^2+^-oxidized LDL are correlated with endothelial dysfunction.

Although immune response against ox-LDL has been suggested by some studies to be associated with the severity of atherosclerosis [18, 19], there has been little data examining the relationship between ox-LDL IgG levels and CAD based on angiography assessment. Therefore, we principally aimed to determine whether IgG antibodies against ox-LDL are associated with CAD. Since conventional cardiovascular risk factors may influence lipid metabolism and immune function, bivariate correlations between serum ox-LDL IgG levels and CAD risk factors and also determinants of anti-ox-LDL levels were sought as ancillary aims of the present study.

## 2. Methods

### 2.1. Study Population

The study population consisted of 63 patients (27 females, 34 males) who were selected from subjects undergoing coronary angiography in the Ghaem Hospital (Mashhad, Iran). Indication of angiography in these patients was for stable angina based on presence of myocardial ischemia in at least one of the following objective tests: exercise test, thallium single photon emission computed tomography (SPECT), and dobutamin stress echocardiography. Coronary angiography was performed using routine procedures. Analysis of the angiograms was performed offline by a specialist cardiologist. The presence of one or more stenoses ≥50% in diameter of at least one major coronary artery (left main, right, left anterior descending, or circumflex artery) was considered as evidence of significant CAD [20, 21]. Patients with stenoses of ≤50% in all major coronary arteries were considered to have a normal angiogram (CAD^−^).

The CAD^+^ patients (i.e., those with at least ≥50% stenosis in at least one major coronary artery) were classified according to the number of significantly affected stenotic vessels into single vessel (1VD; *n* = 7), 2 vessels (2VD; *n* = 13), and 3 vessels (3VD; *n* = 11) disease subgroups. Selected CAD^+^ (*n* = 31; 12 females, 19 males; mean age: 59.39 ± 10.14 years) and CAD^−^ (*n* = 32; 15 females, 17 males; mean age: 58.34 ± 9.68 years) patients were matched for age and gender. Age- and sex-matched healthy volunteers were also recruited as a normal control group (*n* = 24; 6 females, 18 males; mean age: 58.25 ± 9.19 years). The control subjects had never experienced any symptom nor had any signs of CAD. These subjects had no other apparent major disease. Information on smoking, drug use, and family history of CAD was obtained via a questionnaire.

### 2.2. Anthropometric and Other Measurements

For all patients, anthropometric parameters including weight, height, and body mass index (BMI) were measured. Weight was measured with the subjects dressed in light clothing after an overnight fasting using a standard scale. Blood pressure was measured twice while the patients were seated and rested, using a standard mercury sphygmomanometer. The systolic blood pressure was defined as the appearance of the first sound (Korotkoff phase 1), and the diastolic blood pressure was defined as the disappearance of the sound (Korotkoff phase 5) during deflating of the cuff. BMI was calculated as weight (in kilograms) divided by height squared (in square meters).

### 2.3. Blood Sampling and Routine Biochemical Analysis

Blood samples were taken from patients prior to angiography procedure. Following venepuncture, blood samples were collected into Vacutainer tubes and centrifuged at 10,000 ×g for 15 min at 4°C. After separation, aliquots of serum were frozen at −80°C until analysis.

### 2.4. Routine Biochemical Analysis

A full-fasted lipid profile was determined for each subject. Serum lipid and fasting blood glucose (FBS) concentrations were measured by enzymatic methods.

### 2.5. Antioxidized LDL Antibody Assay

IgG autoantibodies against ox-LDL were assayed by ELISA using a commercially available ELISA kit (ox-LDL Antibody ELISA kit, Immundiagnostik AG, Germany), as described by Marroquin et al. [20] and according to manufacturer's specifications. This kit uses individual microplate strips that were coated with native or ox-LDL. The antigen stability was shown to last for at least 4 months. Sera were diluted 1 : 101 prior to the assay.

### 2.6. Statistical Analysis

All statistical analyses were performed using the SPSS for Windows, version 11.5 (SPSS Inc., Chicago, IL, USA). Data were expressed as mean ± SD or median and interquartile range (in case of ox-LDL IgG levels). Group comparisons were performed using one-way ANOVA. Categorical data were compared using Chi-square test. A two-sided *P* value of <0.05 was considered as statistically significant. Bivariate correlations between different parameters and ox-LDL IgG levels were assessed using Pearson's and Spearman's rank correlation coefficients for normally and nonnormally distributed data, respectively. Stepwise multiple linear regression analysis was used to identify determinants of ox-LDL IgG levels. Ox-LDL IgG levels were entered into the model after a square root transformation. Dichotomous (1 = yes/0 = no) variables that were entered into the model included diabetes mellitus, hyperlipidemia, hypertension, and smoking. Height, weight, FBS, waist circumference, hip circumference, high-density lipoprotein (HDL), systolic blood pressure, and number of narrowed vessels (VD) were entered as continuous variables in the same model. Data for age and gender were not included since the patients and healthy subjects were matched for these parameters.

## 3. Results

### 3.1. Demographic Characteristics

The three study groups (CAD^+^, CAD^−^, and control) were matched for age and gender. There was no significant difference in BMI and waist/hip ratio between the groups (*P* > 0.05). However, height, waist circumference, and hip circumference in both CAD^+^ (*P* < 0.05, *P* < 0.05, and *P* < 0.01, resp.) and CAD^−^ (*P* < 0.01, *P* < 0.05, and *P* < 0.01, resp.) groups were significantly lower than those in the control group. Weight was significantly lower in the CAD^−^ versus control group (*P* < 0.01). Mean systolic blood pressure was significantly higher in both CAD^+^ and CAD^−^ groups compared with the control group (*P* < 0.001). No significant difference was observed in diastolic blood pressure, FBS, and CRP between the three groups (*P* > 0.05). Likewise, no significant difference in lipid profile parameters (HDL-C, LDL-C, and triglycerides) was observed among the three groups (*P* > 0.05, Table 1). Demographic parameters were comparable between subgroups of CAD^+^ patients in terms of the number of stenosed vessels (1VD, 2VD, and 3VD) (*P* > 0.05, Table 2). Demographic characteristics of study groups are summarized in Tables 1 and 2.

### 3.2. Ox-LDL IgG Levels in relation to CAD

**Figure 1:**
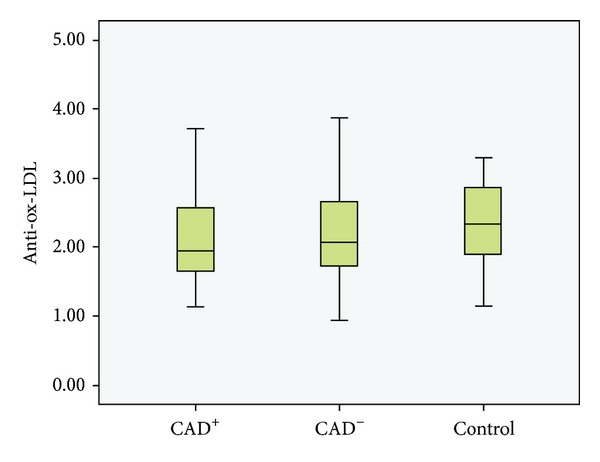
Boxplot of anti-ox-LDL concentrations in CAD^+^, CAD^−^, and control groups. No significant difference was observed among the three groups (*P* > 0.05).

**Figure 2:**
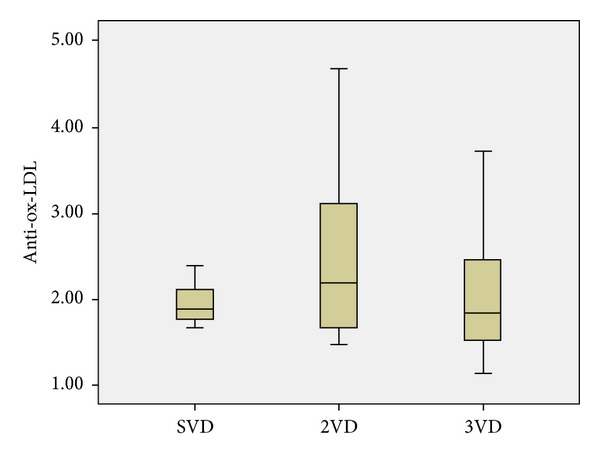
Boxplot of anti-ox-LDL concentration in SVD, 2VD, and 3VD subgroups of CAD^+^ patients. No significant difference was observed among the three groups.

There was no significant difference between CAD^+^, CAD^−^, and control groups regarding anti-ox-LDL concentrations (*P* = 0.83, Figure 1). With respect to the severity of CAD, there was no significant difference in ox-LDL IgG levels between 1VD, 2VD, and 3VD subgroups (*P* = 0.20, Figure 2).

### 3.3. Correlations between Serum Ox-LDL IgG Levels and CVD Risk Factors

**Figure 3:**
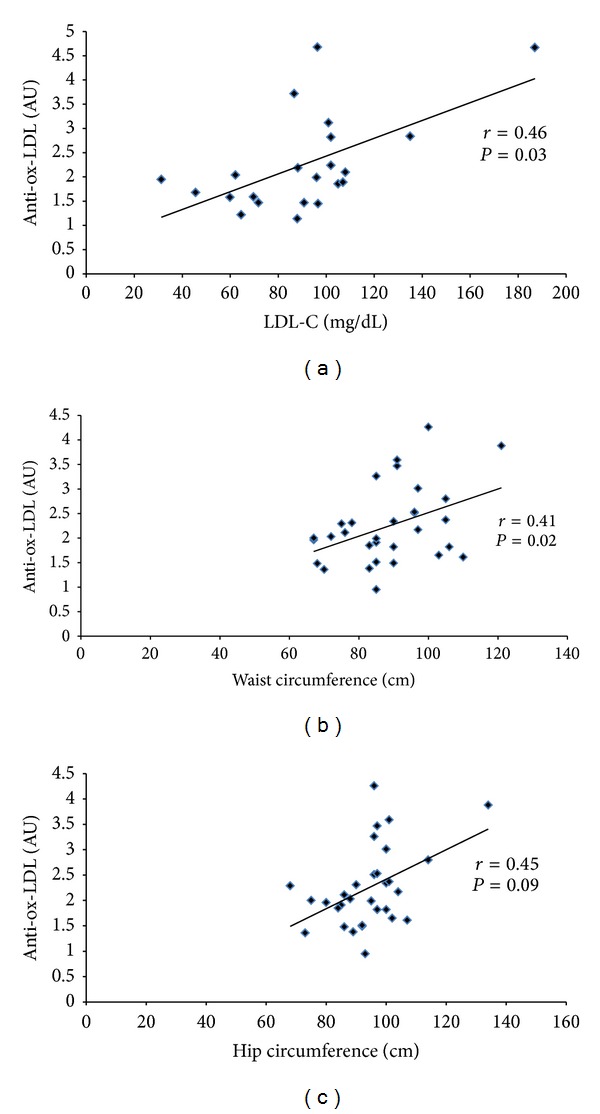
Scatter plots depicting significant correlations between serum ox-LDL IgG levels and (a) LDL-C (in the CAD^+^ group), (b) waist circumference (in the CAD^−^ group), and (c) hip circumference (in the CAD^−^ group). AU: absorbance unit.

In bivariate analyses, serum ox-LDL IgG levels were only significantly correlated with waist circumference and hip circumference (*P* < 0.05 and *P* < 0.01, resp.) in the CAD^−^ group, and LDL-C (*P* < 0.05) in the CAD^+^ group. In the control group, no significant correlation was found between serum ox-LDL IgG levels and CVD risk factors (*P* > 0.05, Table 3 and Figure 3).

### 3.4. Association between Serum Ox-LDL IgG Levels and CVD Risk Factors

In stepwise multiple linear regression analysis, none of CVD risk factors had a significant independent association with serum ox-LDL IgG levels (Table 4).

## 4. Discussion

The present study set out to determine serum ox-LDL IgG levels in CAD^+^ patients with significant (≥50% in luminal diameter) coronary stenosis compared to subjects who are either normal or have stenoses <50%. As a secondary aim, associations between anti-ox-LDL IgG levels and CAD risk factors were explored. The findings did not reveal any significant difference between CAD^+^, CAD^−^, and control groups regarding serum ox-LDL IgG levels. Likewise, there was no association between antibody levels and severity of CAD based on the number of stenosed vessels. In consistence, serum anti-ox-LDL IgG levels were found to be independent of CAD risk factors. Heretofore, several clinical studies have been published on the role of ox-LDL IgG levels in the initiation or progression of CVD. However, data have been controversial. Erkkilä et al. [22] measured levels of autoantibodies against ox-LDL in 415 patients with different manifestations of coronary heart disease (CHD). Anti-ox-LDL antibodies were significantly higher in the group with acute myocardial infarction (AMI) compared to other groups with coronary artery bypass surgery, balloon angioplasty, and acute myocardial ischemia in men, but not in women. Therefore, it was concluded that autoantibodies against ox-LDL are associated with AMI in men. Lehtimäki et al. [23] determined serum levels of antibodies against Cu^2+^-oxidized LDL in 58 patients with angiographically verified CAD and 34 controls without CAD and concluded that elevated levels of anti-ox-LDL are associated with CAD. Inoue et al. [24] measured ox-LDL IgG levels in 108 patients who had angiographically verified CAD and 31 patients who had chest pain but no significant CAD as controls. The ox-LDL antibody levels were found to be higher in patients with multivessel CAD compared to controls. The levels were also higher in patients with unstable angina or in patients with AMI compared to patients with stable-effort angina or old MI. Laczik et al. [25] measured serum ox-LDL IgG levels in a total of 54 patients with acute coronary syndrome (ACS) and 41 matched healthy controls in a prospective study. Higher levels were found in patients with ACS versus controls and it was suggested that such raised levels might have a role in plaque destabilization. Tsimikas et al. [26] investigated the relationship between IgG autoantibodies to ox-LDL and CAD and cardiovascular events in 504 patients undergoing clinically indicated coronary angiography. The results indicated that anti-ox-LDL levels are positively associated with the presence of angiographically determined CAD. Along with the aforementioned studies, there are other reports that support the present findings on the lack of association between anti-ox-LDL levels and CAD: Rossi et al. [27] measured the levels of ox-LDL IgG antibodies in 529 consecutive patients undergoing quantitative coronary angiography for suspected CAD. They found no significant association between anti-ox-LDL levels and CAD severity. Consistently, Virella et al. [28] showed that anti-ox-LDL levels are neither associated with CAD severity nor plasma lipid levels in healthy individuals. Chen et al. [29] examined the association of ox-LDL antibody levels with the severity of CAD in 558 subjects from the Women's Ischemia Syndrome Evaluation Study. Their findings implied that higher ox-LDL IgM levels may provide protection against coronary stenosis. This study is in agreement with that of Rossi et al. [27] who reported an inverse association between ox-LDL IgM levels and presence of angiographically determined CAD. Finally, Che et al. [30] measured the levels of IgG anti-ox-LDL in 154 patients undergoing coronary angiography for suspected CHD and reported that such levels are lower in CHD patients compared to controls.

Taken collectively, it appears that circulating ox-LDL IgG levels are elevated following an acute coronary event, for example, episodes of AMI. This might be due to the impact of plaque rupture which leads to the release and exposure of considerable plaque content of ox-LDL to the circulation, thereby stimulating immune system to produce humoral response. The inconsistent findings on the association of ox-LDL antibody levels with CAD could be attributed to the fact that coronary angiography focuses on the arterial lumen rather than the arterial wall, whilst it has been suggested that rupture-prone (vulnerable) plaques are not those that yield a high degree of stenosis [27]. Moreover, antibodies against ox-LDL have been shown to be heterogenous and IgG and IgM subclasses are known to have different profile of changes following CAD, suggesting another reason for discrepant findings on the anti-ox-LDL status in CAD patients. A number of previous reports which reported elevation of anti-ox-LDL levels following CAD are subject to selection bias [24, 31]. On the other hand a previous large-scale study has provided results very similar to those in the present study [27]. Finally, antibody levels might be affected by ethnicity and a potential explanation for the discrepant findings might be that the profile of immunoglobulin changes differs among ethnicities.

A number of limitations must be acknowledged for the present study. First, only IgG levels against ox-LDL were determined. Besides, the IgGs against ox-LDL are themselves a mixture of antibodies with different specificities, whilst it has been shown that only IgG subclasses against certain epitopes of ox-LDL are implicated in atherogenesis [32]. Second, this study was exclusively conducted among Caucasian subjects and thus was not able to look at a plausible impact of ethnicity on ox-LDL IgG levels. Hence, the findings may not be attributable to CAD patients from author descents. Third, coronary angiography may not be an ideal indicator of arterial wall pathology and rupture-prone atherosclerotic plaques that are more likely to contain significant ox-LDL content.

Given the above limitations, future studies are warranted to assess both IgG and IgM levels against ox-LDL and also quantify different IgG subclasses based on their affinity to ox-LDL. In addition, quantification of ox-LDL antibodies is encouraged to be conducted in patients with acute coronary syndrome because these patients have vulnerable plaques that are known to contain higher amounts of ox-LDL and corresponding antibodies.

It is also recommended to further explore the association between ox-LDL IgG levels and surrogate markers of CAD including carotid intima-media thickness and brachial artery flow-mediated dilation. Finally, any predictive value of ox-LDL IgG levels for CVD endpoints needs to be investigated by prospective studies.

## 5. Conclusions

In summary, findings arising from the present study indicated the lack of clinically significant association between circulating ox-LDL IgG levels, and the presence, severity and conventional risk factors of CAD. With the current available evidence, ox-LDL IgG levels cannot be used for the risk assessment of CAD.

## Figures and Tables

**Table 1:** Demographic and clinical characteristics of CAD^+^, CAD^−^, and control subjects.

	CAD^+^	CAD^−^	Control
Number of subjects	31	32	24
Gender (F/M)	19/12^f^	17/15	6/18
Age	59.39 ± 10.14	58.34 ± 9.68	58.25 ± 9.19
Height	159.07 ± 8.54^a^	157.83 ± 11.99^b^	167.10 ± 7.77
Weight	69.71 ± 11.93	64.40 ± 14.48^b^	75.74 ± 12.67
FBS	138.07 ± 81.75	99.14 ± 28.34	94.18 ± 16.62
BMI	27.39 ± 3.63	26.05 ± 6.87	27.60 ± 4.28
Waist/hip ratio	0.94 ± 0.08	0.94 ± 0.07	0.95 ± 0.16
Waist circumference	88.28 ± 9.54^a^	88.77 ± 13.55^a^	99.16 ± 19.01
Hip circumference	93.88 ± 8.39^b^	94.12 ± 12.44^b^	105.32 ± 15.86
LDL-C	90.67 ± 31.84	107.57 ± 46.99	106.78 ± 20.17
HDL-C	40.60 ± 15.91	38.88 ± 10.88	64.70 ± 88.93
TG	123.69 (47.00–331.00)	157.00 (41.00–352.00)	146.65 (53.00–325.00)
SBP	156.45 ± 30.30^c^	154.08 ± 28.18^c^	123.92 ± 10.59
DBP	77.87 ± 14.47	76.88 ± 14.88	80.35 ± 8.87
Diabetes mellitus (%)	41.9^f^	7.7	12.5
Smoking or addiction (%)	51.6	35.5	12.5
Hypertension (%)	64.5	46.2	41.7
Hyperlipidemia (%)	51.6	38.5	22.2

Values are presented as mean ± SD. FBS: fasting blood sugar; BMI: body mass index; LDL-C: low-density lipoprotein cholesterol; HDL-C: high-density lipoprotein cholesterol; TG: triglycerides; SBP: systolic blood pressure; DBP: diastolic blood pressure. Compared with the control group: ^a^ *P* < 0.05, ^b^ *P* < 0.01, and ^c^ *P* < 0.001; compared with the CAD^−^ group: ^d^ *P* < 0.01, ^e^ *P* < 0.001; comparison between all groups (using Chi-square test): ^f^ *P* < 0.05, ^g^ *P* < 0.001.

**Table 2:** Demographic and clinical characteristics of CAD^+^ subjects with 1, 2, and 3 narrowed vessels.

	SVD	2VD	3VD
Number of subjects	7	13	11
Gender (F/M)	6/1	8/5	5/6
Age	62.14 ± 11.09	58.85 ± 7.90	58.27 ± 12.37
Height	160.00 ± 7.70	158.38 ± 10.46	159.54 ± 6.84
Weight	75.12 ± 7.66	70.00 ± 12.01	67.40 ± 13.26
FBS	167.42 ± 84.27	135.27 ± 91.29	120.60 ± 70.98
BMI	29.07 ± 0.89	27.63 ± 3.76	26.60 ± 4.00
Waist/hip ratio	1.01 ± 0.13	0.93 ± 0.07	0.91 ± 0.05
Waist circumference	94.00 ± 6.21	90.18 ± 11.17	83.90 ± 7.14
Hip circumference	94 ± 12.72	95.90 ± 6.51	91.60 ± 8.72
LDL-C	94.92 ± 17.45	90.23 ± 43.19	88.70 ± 29.34
HDL-C	50.40 ± 20.16	38.95 ± 14.04	36.63 ± 14.42
TG	89 (47.00–128.00)	141.44 (64.00–331.00)	125.22 (60.00–207.00)
SBP	154.28 ± 27.75	166.15 ± 28.14	146.36 ± 33.24
DBP	74.14 ± 13.90	83.46 ± 16.25	73.63 ± 11.20
Diabetes mellitus (%)	57.1	38.5	36.4
Smoking or addiction (%)	57.1	38.5	63.6
Hypertension (%)	71.4	53.8	72.7
Hyperlipidemia (%)	42.9	53.8	54.5

Values are presented as mean ± SD. FBS: fasting blood sugar; BMI: body mass index; LDL-C: low-density lipoprotein cholesterol; HDL-C: high-density lipoprotein cholesterol; TG: triglycerides; SBP: systolic blood pressure; DBP: diastolic blood pressure. No significant difference between three groups (*P *> 0.05).

**Table 3:** Correlations between serum oxLDL IgG levels and CVD risk factors.

	CAD^+^		CAD^−^		Control	
	*r* values	*P* values	*r* values	*P* values	*r* values	*P* values
Age*	−0.11	0.52	0.15	0.40	−0.02	0.90
Height*	−0.27	0.15	0.15	0.41	0.04	0.84
Weight*	−0.12	0.53	0.24	0.19	0.07	0.74
FBS**	0.25	0.19	0.02	0.92	−0.11	0.62
BMI*	0.08	0.68	0.02	0.90	0.18	0.38
Waist/hip ratio*	−0.14	0.47	0.06	0.73	−0.18	0.37
Waist circumference*	0.06	0.77	0.41	**0.02**	−0.16	0.43
Hip circumference*	0.20	0.32	0.45	**0.009**	0.06	0.75
LDL**	0.46	**0.03**	0.06	0.77	−0.30	0.21
HDL**	0.10	0.65	−0.30	0.16	−0.29	0.22
TG*	−0.02	0.91	0.16	0.43	0.007	0.97
SBP*	0.12	0.49	0.13	0.50	0.05	0.84
DBP*	0.14	0.43	0.26	0.19	0.27	0.34

*Pearson's correlation analysis.

**Spearman's correlation analysis.

**Table 4:** Association between serum anti-oxLDL titres and CVD risk factors in stepwise multiple regression analysis.

Covariate	*β*	*P*	95% confidence interval
Age	−.162	.616	−.096–0.60
FBS	.402	.290	−.007–0.020
LDL-C	.090	.759	−.021–0.028
HDL-C	−.290	.349	−.085–0.034
TG	.005	.990	−.016–0.016
SBP	.136	.693	−.022–0.031
DBP	−.388	.389	−.099–0.043
DM	.227	.546	−1.676–2.938
Smoking	.105	.699	−.482–0.684
HTN	.360	.275	−.720–2.210
HLP	−.123	.592	−1.337–0.815
BMI	−.651	.615	−.599–0.378
Waist hip ratio	−2.085	.430	−105.209–49.503

FBS: fasting blood sugar; BMI: body mass index; LDL-C: low-density lipoprotein cholesterol; HDL-C: high-density lipoprotein cholesterol; TG: triglycerides; SBP: systolic blood pressure; DBP: diastolic blood pressure; HTN: hypertension; HLP: hyperlipidemia; BMI: body mass index.
